# The reliability, validity, and sensitivity of the Edmonton Frail Scale (EFS) in older adults with foot disorders

**DOI:** 10.18632/aging.202140

**Published:** 2020-12-21

**Authors:** Emmanuel Navarro-Flores, Ricardo Becerro de Bengoa Vallejo, Marta Elena Losa-Iglesias, Patricia Palomo-López, César Calvo-Lobo, Daniel López-López, Eva Maria Martínez-Jiménez, Carlos Romero-Morales

**Affiliations:** 1 Faculty of Nursing and Podiatry, Department of Nursing, University of Valencia, Frailty Research Organized Group (FROG), Valencia, Spain; 2 School of Nursing, Physiotherapy and Podiatry, Universidad Complutense de Madrid, Madrid, Spain; 3 Faculty of Health Sciences, Universidad Rey Juan Carlos, Madrid, Spain; 4 University Center of Plasencia, Universidad de Extremadura, Plasencia, Spain; 5 Research, Health and Podiatry Group, Department of Health Sciences, Faculty of Nursing and Podiatry, Universidade da Coruña, A Coruña, Spain; 6 Antonio Nebrija University, Madrid, Spain; 7 Faculty of Sport Sciences, Universidad Europea de Madrid, Villaviciosa de Odón, Madrid, Spain

**Keywords:** frailty, elderly, foot deformities, foot diseases, foot disorders

## Abstract

The Edmonton Frail Scale (EFS) is an index employed to measure alterations related to frailty. The main objective in this research was to develop the EFS short-form (EFS-SF) and to evaluate its validity, reliability, and sensitivity to predict frailty disability outcomes in elderly patients with foot disabilities.

Results: Exploratory factor analysis (EFA) of the EFS-SF revealed the presence of three components, as in the original EFA. There were significant differences (*p* < 0.05) in the study population for several of the EFS and 5-item FRAIL scale indicators. The highest correlation (Pearson *R* = 0.871; *p* < 0.001) was found for the first component of the EFS-SF. Finally, the Cronbach alpha was 0.864 which indicated a high level of internal consistency.

Conclusion: The EFS-SF is a reliable and valid instrument to measure frailty in patients with and without foot disabilities.

Method: A cross sectional descriptive study was carried out. The study population was aged over 60 years (*n* = 66) and comprised 29 men and 37 women. Frailty disorders were registered by using the EFS, 5-item FRAIL scale, and the Geriatricians’ Clinical Impression of Frailty (GCIF) scale. EFA was employed to locate potential constituents of the EFS, with scores ranging from 0.596 to 0.946 for each of the sub scales: (1) cognitive and general health status; (2) medication and nutrition status; and (3) functional and physiological status, thus revealing that the EFS-SF comprised three components, a reduction compared to the nine in the original EFS.

## INTRODUCTION

Chronic diseases in aging populations, such as diabetes mellitus, osteoarticular disabilities, and heart processes may produce a state of vulnerability and frailty syndrome. The latter is defined as a gradual process characterized by several psychological, biological, and social aspects which together cause a decline in patient health status [1]. Chronic degenerative disorders can also produce some alterations in mental and general health. In fact, frailty syndrome decreases step velocity and increases the risk of falls due to gait changes [2–4]. Moreover, frailty conditions also influence health-related quality of life (QoL) [5], especially in older adults. We found no evidence in the scientific literature that the degree of frailty in elderly individuals is related to sex.

As the prevalence of frailty syndrome is growing, it also appears that more elderly people require specific foot care which, when not effectively managed, can develop into major problems [6] and consequently, increase their risk of falls [7, 8] and of chronic fatigue related to foot alterations [9]. The Edmonton Frail Scale (EFS) is an index used to measure alterations related to frailty. The EFS assesses nine subscales (1) cognition; (2) general health status; (3) functional independence; (4) social support; (5) medication use; (6) nutrition; (7) mood; (8) continence; and (9) functional performance (in 11 items). The highest possible score is 17 points and corresponds to the highest degree of frailty [10]. The degree of frailty is assessed by scoring it, with 0 to 4 points representing the absence of frailty, scores of 5 to 6 indicating vulnerability, 7 to 8 corresponding to low-level frailty, 9 to 10 representing moderate frailty, and scores exceeding 11 indicating severe frailty [11]. To date, no studies have managed to reduce the number of EFS subscales or to correlate them to other frailty scores.

Thus, the purpose of this current work was to reduce the number of subdomains in the EFS, thus converting it into a short form (EFS-SF) which can be used to measure foot-related problems in older adults. Our aim was to develop implementable strategies for clinical professionals to help them reduce the exposure of elderly individuals to risk factors and thus, prevent complications [12].

We correlated the EFS with the 5-Item Fatigue, Resistance, Ambulation, Illnesses, and Loss of Weight (FRAIL) Score and the Geriatricians’ Clinical Impression of Frailty (GCIF) scale, which has also been used in a cohort older acute patients [13], in order to reduce the nine sub-domains of the original EFS to three in the EFS-SF. We then evaluated its validity, reliability, and sensitivity to predict frailty disability outcomes in elderly patients with foot disabilities. We hypothesized that the EFS-SF would be a reliable and valid instrument to measure the extent of frailty in older individuals with foot disorders.

## RESULTS

### Sociodemographic data

We studied a population of 66 adults with a mean age of 76.80 ± 9.99 years. The study participants included 37 (56.10%) women and 29 (43.90%) men. Their sociodemographic data is summarized in Table 1. There were no significant sociodemographic differences according to sex (*p* > 0.05) for age or body mass index (BMI), although the mean weight and height was higher in men compared to women (*p* > 0.05). The birth date, height (cm), weight (kg), and BMI anthropometric variables showed a normal distribution (*p* > 0.05), while all the items on the 5-item FRAIL Score instrument and EFS scale had a non-normal distribution (*p* < 0.05).

**Table 1:** Descriptive and sociodemographic data for the study sample.

**Demographic and descriptive data**	**Total Group *N* = 66 Mean ± *SD***	**Female *n* = 29 Mean ± *SD***	**Male *n* = 37 Mean ± *SD***	***P*-value**
Age (Years)	77.47 ± 10.69 (74.54–80.40)	79.07 ± 10.74 (75.16–82.98)	75.36 ± 10.50 (70.98–79.75)	0.224
Weight (kg)	62.47 ± 12.08 (59.16–65.78)	58.31 ± 12.44 (53.78–62.84)	67.95 ± 9.25 (64.09–71.82)	0.004
Height (m)	1.61 ± 0.08 (74.54–80.40)	1.57 ± 0.07 (1.54–1.59)	1.65 ± 0.07 (1.62–1.68)	0.000
BMI (Kg/m^2^)	24.19 ± 3.96 (23.10–25.27)	23.67 ± 4.30 (22.10–25.24)	24.87 ± 3.42 (23.45–26.30)	0.286

BMI: body mass index; the mean ± standard deviation (*SD*), and range (min–max) are shown. Probabilities were calculated using Student *t*-tests for independent samples. In all the analyses, *p* < 0.05 (with a 95% confidence interval), was considered statistically significant.

### The EFS versus the 5-item FRAIL Score and GCIF for predicting frailty

**Figure 1:**
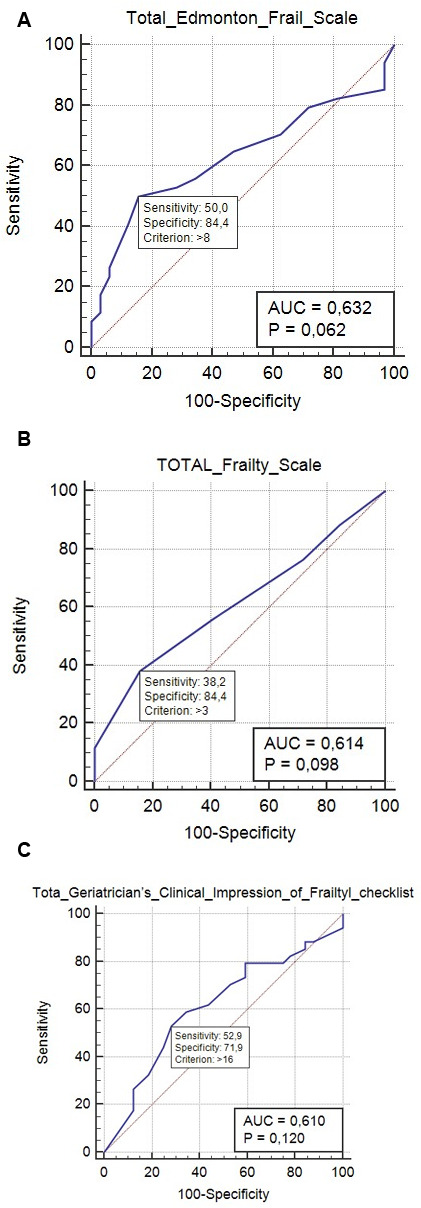
(**A**) Receiver operating characteristic (ROC) curve for the Edmonton FRAIL Score (EFS) scale for predicting frailty; the optimal prediction point (calculated as the Youden diagnosis index) was 0.632, with a sensitivity of 50% and a specificity of 84%. (**B**) ROC curve depicting the 5-item FRAIL Score for predicting frailty; the optimal prediction point was 0.614 (Youden index), with a sensitivity of 38.2% and a specificity of 84.4%. (**C**) ROC curve for the>Geriatricians’ Clinical Impression of Frailty (GCIF) checklist for predicting frailty; the optimal prediction point (Youden index) was 0.610, with a sensitivity of 52.9% and a specificity of 71.9%.

To compare the efficacy for predicting frailty in different individuals, we calculated the area under the curve (AUC) for each scale. For the EFS, the AUC was 0.632 (*p* = 0.062), with a sensitivity of 50.0% and specificity of 84.4%. In contrast, the AUC for the (GCIF) was 0.610 (*p* = 0.120), with a sensitivity of 52.9% and a specificity of 71.9%. The AUC for the EFS was higher than that of GCIF, as shown in Figure 1, suggesting that the EFS was a better predictive tool.

### Factor structure

The Kaiser–Meyer–Olkin value for the EFS-SF was 0.874 and it comprised three components with a factor loading > 0.4 which accounted for a total variance of 73.02% which was comparable to that of the original EFS subdomains.

### Correlations between the EFS component factors

Pearson coefficients were calculated to determine the correlations between the EFS component factor scores (Table 2). There was significant correlation between the overall value and the three aforementioned components, which was especially strong for the first factor, accounting for 43.785% of the factorial model; for factors 2 and 3 this was 16.543%, and 12.374%, respectively).

**Table 2:** Correlation between the three Edmonton Frail Scale-Short Form (EFS-SF) subscales.

**Correlations**		**Factor 1: cognition and general health status, *R* (P)**	**Factor 2: medication and nutrition status, *R* (P)**	**Factor 3: functional and physiological status, *R* (P)**
Total_EFS	Pearson correlation	0.871 (0.000)	0.326 (0.007)	0.308 (0.012)
	% of variance	43.875	16.543	12.374

The Pearson correlations (R) and associated *P*-values are shown. In all the analyses, *p* < 0.05 with a 95% confidence interval was considered statistically significant. EFS: Edmonton Frail Scale.

### Reliability analysis

The inter-rater reliability for the total EFS was strong (*R* = 0.784; *p* < 0.001). The variables were divided into 8 common items and these showed adequate correlations between the scores for each variable as well as the overall scores when considering a statistical significance of *p* < 0.05. The Cronbach α was determined to assess the internal consistency (IC) of each subdomain of the EFS-SF (Table 3). The intraclass correlation coefficient (ICC) was used to indicate reliability. Exploratory factor analysis (EFA) by varimax rotation (requiring a value of 0.874 for the loading of each factor) was employed to identify possible subscales.

**Table 3:** Exploratory factor analysis results.

**Edmonton Frail Scale item**	**Components**		
	**Factor 1: cognition and general health status**	**Factor 2: medication and nutrition status**	**Factor 3: functional and physiological status**
1) cognition and general health status, Cognition ITEM 1_EFS CLOCK TEST	0.946*	0.035	0.025
1) cognition and general health status, General health status ITEM 2A_EFS	0.890*	0.210	−0.091
1) cognition and general health status, General health status ITEM 2B_EFS	0.874*	0.238	0.116
1) cognition and general health status, Social support ITEM 4_EFS	0.701*	0.118	0.094
1) cognition and general health status, Continence ITEM 8_EFS	0.596*	0.424	−0.032
2) medication and nutrition state Nutrition ITEM 6_EFS	0.556	0.741*	0.333
2) medication and nutrition state Medication use ITEM 5A_EFS	0.242	0.732*	0.030
2) medication and nutrition state Medication use ITEM 5B_EFS	0.345	0.723*	−0.210
2) medication and nutrition state Functional performance ITEM 9_EFS TIME UP AND GO	0.002	−0.613*	0.162
3) functional and physiological state Mood ITEM 7 Edmonton Frail Scale	−0.056	−0.055	0.921*
3) functional and physiological state Functional independence ITEM 3Edmonton Frail Scale	0.546	0.340	0.596*

Rotation Method: Varimax with Kaiser normalization.

The validity of the EFA-SF was evaluated by correlating the total score for each participant with their 5-item FRAIL and GICF scores (Table 4). The total mean score for each EFS sub-scale was different. The variance of the overall mean participant scores was compared in each EFS domain using ANOVAs. To evaluate the mean differences and show the sensitivity of the clinometric tool, the mean differences in different frailty test scores both with and without foot disorders were compared by ANOVA, after first having tested for homogeneity of the variances.

**Table 4:** Reliability statistics for the Edmonton Frail Scale-Short Form (EFS-SF).

**EFS-SF domains**	**Overall item statistics**			
	**Scale mean if item deleted**	**Scale variance if item deleted**	**Corrected item-total correlation**	**Cronbach alpha if item deleted**
Cognition ITEM 1Edmonton Frail Scale CLOCK TEST	6.201	12.843	0.840	0.844
General health status ITEM 2AEdmonton Frail Scale	6.308	13.047	0.822	0.846
General health status ITEM 2BEdmonton Frail Scale	6.1527	1.,492	0.850	0.840
Functional independence ITEM 3Edmonton Frail Scale	6.347	12.465	0.681	0.856
Social support ITEM 4Edmonton Frail Scale	6.472	14.054	0.606	0.861
Medication use ITEM 5A Edmonton Frail Scale	6.371	14.611	0.543	0.866
Medication use ITEM 5A Edmonton Frail Scale	6.426	14.682	0.513	0.867
Nutrition ITEM 6 Edmonton Frail Scale	6.317	15.139	0.392	0.874
Mood ITEM 7 Edmonton Frail Scale	6.431	15.745	0.195*	0.884*
Continence ITEM 8 Edmonton Frail Scale	6.571	14.299	0.654	0.860
Functional performance ITEM 9 Edmonton Frail Scale TIME UP AND GO	5.965	14.840	0.310	0.882

*Low correlation item for which the Cronbach α increased if item was deleted.

## DISCUSSION

Frailty is now measured by clinical geriatricians as part of standard clinical practice. Frailty scores may be able to predict disorders related to aging, the risk of falls, weight loss, or decreased gait speed [1, 13, 14]. In this current research we evaluated ability of three indices, the GICF, 5-item FRAIL Score, and EFS to determinate the degree of frailty among elderly patients with foot disorders. We found that a high percentage of these patients were frail, perhaps in relation to osteoarticular conditions.

The 5-item FRAIL Score is an index comprising five categories which was developed using a self-administered construct [1]. The five categories correspond to (1) fatigue; (2) resistance; (3) ambulation; (4) illnesses; and (5) loss of weight. Fatigue was determined by inquiring about the individual’s feeling of exhaustion; resistance was evaluated according to the patient’s ability to climb stairs; ambulation was considered when the individual was able to walk; illnesses corresponded to the presence of at least 5 pre-defined illnesses from a total of a possible 11 (e.g., cardiovascular disabilities, diabetes, etc.), and weight loss was determined if the individual had experienced a weight reduction of 5% in the 12 months prior [15]. The items have binary yes/no answers, with 1 point being assigned to positive responses on a scale of 0 to 5. Individuals are scored as robust (0 points), pre-frail (1–2 points), or frail [≥ 3 points].

As previously mentioned, the original EFS assesses nine subscales. In comparison, the EFS-SF has three sub domains comprising nine items. Furthermore, the EFS-SF better correlates than EFS (*r* = 0.884 vs. *r* = 0.886). The EFS-SF correlated well with results from older adults with foot disorders and can be used to predict frailty syndrome. Moreover, it has the advantage that it reduces the nine original subdomains to only three: (1) cognition and general health status; (2) medication and nutrition status; and (3) functional and physiological status; questions related to mood and functional independence were excluded.

A similar number of variables were used to construct the 5-item FRAIL and GCIF scales. Therefore, even though measurement of some of their cut-offs and deficits is unclear [13], it was useful to compare their predictive values to the EFS-SF because they contain an appropriate number of questions to properly evaluate individuals with foot disorders. Moreover, some items referring to mood or gait can be reduced in the EFS-SF because they do not show adequate validity when they are grouped. The current application of the EFS instrument to assess items related to frailty (such as walking, fatigue, or weight loss) is reliable. Therefore, it is more useful for evaluating frailty terms than other frailty indices such as the Frailty Trait Scale (FTS) [16] or Tilburg Frailty Indicator (TFI) [17].

Because of the frequency of the presentation of frailty factors, especially in older adults, adequate outcomes are required to measure the degree of frailty. Previous research has examined gait parameters [4], showing that frailty related to biomechanical parameters like gait speed present lower indices and correspond to higher frailty scores among females versus males [14, 18]. Furthermore, in agreement with our own results, certain disabilities have been associated with an increased risk of frailty [19, 20]. Thus, balance and walking disorders are particularly predictive of frailty symptoms, and specifically, women with foot disorders exhibited higher frailty scores compared to their male counterparts. The only exception was the mood domain, which also seemed to be related to older adults suffering from a foot disorder [5, 21].

Our findings showed that the GCIF and EFS had good efficacy to predict frailty scores. Furthermore, the predictive validity of the GCIF was higher than that of EFS and 5-item FRAIL score. Prior research grouped the degree of frailty into minor, moderate, and high levels [12, 13], which can be tested in populations with frailty or suspected of having a frailty condition, such as those included in this present research. The original EFA was stronger in some domains than in others and so we proposed reducing the number of items included in this scale.

Although there are differences between the item subdomains, their inter-item reliability was good, with an ICC > 0.7. The validity of EFS-SF also strongly correlated (*R* > 0.9) with the overall scores for the GCIF and 5-item FRAIL Score. Thus, the EFS-SF is a reliable clinimetric tool. The Cronbach α for all the items included in this study was comparable with the original EFS. Furthermore, the ICC showed strong clinimetric tool test–retest reliability. In addition, the EFA results showed that the factorial analysis identified item correlations and EFS-SF subdomains. However, the optimal point for predicting frailty using the GCIF was not reported. This study showed that when the GCIF exceeded 17 points, the probability of frailty was higher. Moreover, using a reduced EFS scale can be useful to measure the degree of frailty, and so we propose reducing the original nine subdomains to three domains in the EFS-SF.

Frailty results can be helpful in specific interventions, even for treatments for chronic diseases for which physical activity and nutritional status assessment are prescribed [1]. The GCIF was negatively correlated with the mood value for the EFS (R = −0.018, p < 0.005), while the association between the GCIF and 5-item FRAIL Score was not significant (p = 0.170). However, the reference to daily activities on both the 5-item FRAIL score and the EFS correlated with the GCIF score. Regarding concurrent validity, the 5-item FRAIL scale resistance domain score showed the poorest correlations and so the final version of the EFS-SF does not contain a specific subscale for intensive physical exercise. The highest correlations were found for cognitive and general health domains. This may be because certain EFS domains were based on GCIF subscales. However, this is the first research to measure and compare the sensitivity of the GCIF, 5-item FRAIL scale, and original EFS [10, 13, 22] Thus, future research should consider every risk factor associated with frailty syndrome. Both the EFA [14, 23] and the GCIF have been used to determine the fragility score, in the latter case in a cohort of older acute patients [13].

Regarding the limitations of this work, this study was limited to a sample in in Spain and so future work should consider samples from several other countries and cultural contexts in order to corroborate the usefulness of the EFS-SF. Moreover, our study only considered the sensitivity, validity, and reliability of the EFS-SF in an elderly Spanish population with foot problems. Although gait disorders and balance alterations leading to an increased risk of falls are very common in frail individuals [2, 4], work should also be carried out in other samples to assess the frailty index in, for example, frail men who live alone—given that this population usually have higher scores as the consequence of psychosocial disabilities [23–25].

Moreover, population selection could have been another source of bias in this work. Therefore, future work should analyze a randomized study population. In addition, although we employed the EFS, other frailty questionnaires such as the Fried or Tilburg scales are available and have also been used to measure the degree of frailty [1, 26, 27] and so, should also be studied in future research. Finally, in this current work we did not correlate the influence of different foot disabilities, congenital alterations, acquired diseases, traumas, or chronic diseases, because our population sample was not adequately adjusted for this purpose. Thus, these comparisons should be made in future studies.

## CONCLUSIONS

The EFS-SF is a useful scale with an adequate sensitivity, reliability, and validity to grade Spanish populations of older adults into five different frailty-degree categories. This present study provided new evidence that a reduced-items version of the EFS, the EFS-SF instrument, shows increased consistency and is a self-administered test that can be reliably be used in clinical research and in medical evaluations to assess the degree of frailty in patients with and without foot pain.

## MATERIALS AND METHODS

### Participants

This research was carried out in Spain between November 2019 and January 2020 in 66 adults aged more than 60 years enlisted at a geriatric hospital [12]; we obtained signed consent to participation from all of the individuals enrolled. This observational, descriptive study was developed employing the STROBE guidelines [28]. This work was approved by the Human Research Ethics Committee at the University of Extremadura (reference code 1/2020).

### Inclusion criteria

(1) Adults aged over 60 years; (2) A history of foot pain in the 12 weeks prior. To recruit the study population, we held informative talks at the center for the elderly where we invited the center’s users to participate in this research study. When a potential participant expressed interest, we conducted a cognitive function assessment interview to determine if they were eligible. We subsequently explained the research procedures in detail to the study population.

### Exclusion criteria

(1) Significant cognitive disability (individuals who were unable to respond to the questions on their own or who would not able to participate in a normal way); (2) Patients who refused to participate in the study or did not provide their signed consent prior to the start of the work.

### Evaluation of frailty

The questions used in this work enquired about the participants’ general state of health, socio-demographic characteristics (sex, birth date, body-mass index), and chronic pathologies (e.g., physiological disorders, osteoarticular diseases, cardio vascular disease, etc.). Specific questions about foot disabilities, such as having received orthopedic treatments or toe deformities, were also assessed. To be diagnosed with frailty, an individual must present three of the principal five characteristics of frailty: (1) weakness; (2) sluggishness; (3) weight loss; (4) low levels of physical activity; and (5) fatigue. Patients with some of these characteristics can be classified as prefrail while conversely, robust individuals do not exhibit any of these qualities [19].

Patients completed the EFS to evaluate nine frailty subscales: As previously described, patients completed the EFS to evaluate nine frailty subscales, with scores ranging from 0 to 17 points [10, 12], scored from 0 to 17 points. This questionnaire can be completed in a few minutes. The EFS classifies patients into one of three levels, with higher scores corresponding to a higher degree of frailty. Patients with scores lower than 5 points were classified as not frail; those who scored between 12 and 17 points were classified as prefrail; the most frail population obtained 6 to 11 points.

The study population also completed the 5-item FRAIL Score [22] which is divided into five subdomains: (1) fatigue; (2) resistance; (3) ambulation; (4) illnesses; and (5) loss of weight. The results from this index range from 0 (best) to 5 (worst), with scores between 3 and 5 classified as fragile, 1 or 2 points classified as pre-fragile, and individuals with a score of 0 considered non-fragile.

### Sample size calculation

The sample size of the study population was calculated and estimated using two series-model correlation tests with G*Power 3.1.9.2 software (G*Power^©^; Dusseldorf University; Germany). In addition, a moderate correlation coefficient of *r* = 0.4 [29], a two-tailed hypothesis, an error of α = 0.05, with a confidence interval of 95% and β error = 20% and power analysis of 1−β = 0.80 were considered. Thus, a sample size of 44 individuals was considered appropriate for this work.

### Statistical analysis

Using the Shapiro–Wilk test, whole variables were considered normally distributed when *p >* 0.05. With respect to quantitative variable outcomes, non-normally distributed data were described as the median, interquartile range (IR), and minimum and maximum (range) values. Normally-distributed data were described using the mean, standard deviation (*SD*), and range values. To compare quantitative results between men and women for the different instrument subdomains (EFS, GCIF, and 5-item FRAIL scale) independent Student *t*-tests were carried out while non-normal results were analyzed using Mann–Whitney U tests. For categorical variables, Chi-squared tests were used to check for significant differences among the observed frequencies. Non-parametric tests were used to identify any correlations between the subdomains of the 5-item FRAIL Scale subscales [5, 16, 18] and the EFS. Spearman’s correlation coefficients (*r*s) were determined and were qualified as low (*r*s ≤ 0.40), moderate (0.41 ≤ *r*s ≥ 0.69), or high (0.70 ≤ *r*s ≥1.00). The inter-rater reliability and Cronbach α coefficient for the reliability of the scale were also calculated.

To compare metrics and validate the EFS, the 5-item FRAIL scale and GCIF were also administered to all the participants. We performed receiver operating characteristic (ROC) curve analysis to describe the score allocation for the 5-item FRAIL and EFS to predict the degree of frailty. Next, the area under the curve (AUC) was calculated, with the optimal predictive amount being defined by the highest Youden diagnosis index, which is equivalent to the variation between the sensitivity and specificity. The higher the Youden index cut-off point, the higher the positive predictive value. We calculated Pearson correlation coefficients to correlate the total scores for the EFS domains and 5-item FRAIL scale and GCIF scores.

For all of the analyses, statistical significance was considered at *p* < 0.05 with a 95% confidence interval (CI). The statistical analyses were carried out using SPSS software (V.26.0, IBM Corp., Armonk, NY).
